# Helminth Protein Vaccine Induced Follicular T Helper Cell for Enhancement of Humoral Immunity against *Schistosoma japonicum*

**DOI:** 10.1155/2013/798164

**Published:** 2013-11-06

**Authors:** Jingyao Zhang, Wenjuan Gao, Qirui Guo, Bobo Huang, Bin Wang, Guoliang Xia, Youmin Kang

**Affiliations:** ^1^State Key Laboratory for Agro-Biotechnology, College of Biological Science, China Agricultural University, Beijing 100193, China; ^2^Key Laboratory of Medical Molecular Virology of MOH and MOE, Fudan University Shanghai Medical College, Shanghai 200032, China

## Abstract

Protein vaccines combined with adjuvants have been widely used to induce immune responses, especially the humoral immune response, against molecular targets including parasites. Follicular T helper (Tfh) cells are the specialized providers of B-cell help, however, the induction of Tfh cells in protein vaccination has been rarely studied. Here, we report that the *Schistosoma japonicum* recombinant protein (SjGST-32) combined with tacrolimus (FK506) augmented the induction of Tfh cells, which expressed the canonical markers CXCR5, BCL6, and IL-21, and enhanced the humoral immune responses in BALB/c mice. Furthermore, the expression of IL-21R on germinal center (GC) B cells and memory B cells increased in immunized mice, which indicated that IL-21 from the induced Tfh cells interacted with IL-21R for activation of B cells and maintenance of long-lived humoral immunity. Our results suggest that helminth protein vaccine combined with FK506 induces Tfh cell for stimulating humoral immune responses and inducing long-lived humoral immunity.

## 1. Introduction

Antibody production is critical for pathogen clearance. Humoral responses to most proteins are strictly T cell-dependent (TD) [1]. Tfh cell is a subset of effector T cells that helps the development of Ag-specific B-cell responses in GCs. Tfh cells depend on CXCR5 to localize in the follicular regions of lymphoid organs and maintain stable contact with Ag-primed B cells [2, 3]. In particular, Tfh cells secrete the cytokine IL-21, which drives the growth, differentiation, and isotype switching of B cells [4]. In some cases, Tfh cells have been shown to make the Th2 signature cytokine IL-4 [5–7]. Tfh cells are also characterized by expression of the inducible costimulatory receptor ICOS [8] and the inhibitory receptors PD-1 [9] and B and T lymphocyte attenuator (BTLA) [10]. Furthermore, Tfh cells express high levels of Bcl6 which was recently identified as a master regulator of Tfh differentiation [10].

Schistosomiasis is a chronic parasitic disease that affects more than 200 million people worldwide, mostly in developing countries. Schistosome are also an important pathogen for several domestic animal species and causes economic losses in endemic areas [11]. Currently, the development of a vaccine is the obvious practical measure for disease control. The use of irradiated cercariae for vaccination has been the best animal model described, leading up to 90% or more protection against challenge infection [12]. However, culture the parasitic pathogen in large amounts for vaccine preparation is completely impractical. Hence, the identification of relevant immunogens is imperative for the development of an anti-Schistosome vaccine [4, 13]. Many potential anti-schistosome vaccine candidates have been identified in animal models and human studies. The *Schistosoma japonicum* soluble adult worm extract (SWAP), Sj97, Sj22.6, and Sj67 were used in a population from Leyte and The Philippines after treatment with Praziquantel. The cytokine responses to *Schistosoma japonicum* were tested. The *Schistosoma* antigen Sj97-based vaccine generated a polarized Th-2 response as central in human resistance to schistosoma [14]. Th2 cytokines including IL-4 were found to be absolutely necessary for resistance to primary and/or secondary schistosomiasis in mice [15]. Various approaches are in progress recently in order to improve the potency of the immunogens, particularly with respect to the choice of a better adjuvant. A DNA vaccine encoding the SjGST-32 gene, which consists of fused SjGST and Sj32 genes, combined with levamisole (LMS) as an adjuvant stimulated SjGST-32 protein-specific cellular immune responses [16], but the efficacy needs to be proved. 

FK506 is a widely used immunosuppressant for treating allergies, autoimmune diseases, and transplant rejection. FK506 is known to suppress the activation and the production of IL-2 by T cells [17]. Recently, evidence has accumulated indicating that FK506 can affect the development and antigen presentation of dendritic cells (DCs), as well as the DC:T cell interaction [18] and FK506 as adjuvant of DNA vaccines induced regulatory T cells (Treg) and prevented Multiple Sclerosis (MS) and autoimmune ovarian disease [19, 20]. Our previous results showed that FK506 as an adjuvant for OVA protein enhanced humoral immune responses [21], while it did not induce Th17 and Treg cell populations. Here, we investigate the adjuvant effect of FK506 on the humoral immune response to a recombinant protein of *Schistosoma japonicum* (SjGST-32). We find that FK506 induces Tfh cells which secrete IL-21 for activation and proliferation of B cells and also the generation of memory B cells.

## 2. Materials and Methods

### 2.1. Animals and Reagents

Female BALB/c mice (6–8 weeks) were purchased from Animal Institute of Chinese Medical Academy (Beijing, China) and received pathogen-free water and food for maintenance. All animal protocols [no. 20130101] were approved by the Animal Welfare Committee of China Agricultural University. The animals were housed with pathogen-free food and water under a 12 h light-cycle condition. FK506 from Astellas Ireland Co., Ltd. (Ireland) was described previously [19]. CFSE was from Molecular Probes (Eugene, OR). All antibodies for FACS analysis were from eBioscience (San Diego, CA).

### 2.2. The Expression of Schistosoma Japonica Recombinant Protein

The plasmid and the recombinant protein SjGST-32 expression were described previously [16, 22]. The SjGST and Sj32 genes forming one open reading frame were digested from plasmid VR1012-SjGST-32 and were subcloned into the pGEX-3X-1 (Invitrogen Inc., USA) for the expression of *Schistosoma japonicum* recombinant protein SjGST-32. The recombinant protein SjGST-32 was purified by glutathione-sepharose chromatography.

### 2.3. Immunization

Mice were randomly divided into four groups (*n* = 9) and each mouse was immunized with 100 *μ*g recombinant protein SjGST-32 with 10 *μ*g FK506 subcutaneously in back, while mice immunized with SjGST-32, FK506, or saline only was considered as controls. All mice were boosted on day 14 with the same dose of FK506 and SjGST-32.

### 2.4. ELISA

Seven days after the second immunization, serum was collected and analyzed by ELISA. All ELISA plates were coated with the SjGST-32 protein (5 *μ*g/mL) and blocked with 5% milk. The serially diluted serum was added. As secondary antibodies, 100 *μ*L of horseradish peroxidase-labeled goat anti-mouse IgG, or IgG1 (Sigma, St. Louis) were added and incubated at 37°C for 1 h. The dilutions for these antibodies were at 1 : 3000 for IgG and IgG1. After five washes with PBST, 10 mg of TMB tablet (Sigma, St. Louis) were dissolved in 0.025 M phosphate-citrate buffer and 50 *μ*L of the resulting solution were added to each well. The reaction was stopped by addition of 0.2 M H_2_SO_4_. OD at 450/620 nm was analyzed with a plate reader (Magellan, Tecan Austria GmbH). Titer values were assigned as the highest dilution at which the optical density was 2 SDs higher than the OD produced by the serum of naive mice at the equivalent dilution. The mean of titers were calculated using log conversion for each dilution.

### 2.5. B-Cell Proliferation

Seven days after the second injection, splenocytes of immunized mice were prepared and B cells were purified via positive selection by magnetic cell sorting (Mouse B Cell Isolation Kit, Order no: 130-090-862, Miltenyi Biotec Inc., USA). The purity was above 93%. The purified B cells were labeled with CFSE (1 *μ*M) and added into flat-bottom 96-well plates for the B-cell proliferation assay, with the SjGST-32 protein (5 *μ*g/mL) or LPS (1 *μ*g/mL) as stimulators. The samples were cultured for 3 days at 37°C and analyzed by FACS.

### 2.6. Staining of Surface and Intracellular Molecules for FACS Analysis

For surface staining, splenocytes were blocked with anti-CD16/32 mAb, and immunostained with anti-mouse B220-PECy5 (Clone: RA3-B2), GL-7-FITC (Clone: GL-7), and CD95-APC (Clone: 15A7) for GC B cell analysis; with anti-mouse B220-PECy5 (Clone: RA3-B2), GL-7-FITC (Clone: GL-7), CD95-APC (Clone: 15A7), and IL-21R-PE (Clone: eBio4A9) mAbs for IL-21R expression on GC B cell analysis; with anti-mouse B220-PECy5 (Clone: RA3-B2) and CD27-FITC (Clone: LG-7F9) for memory B-cell analysis; or with anti-mouse B220-APC (Clone: RA3-B2), CD27-FITC (Clone: LG-7F9), and IL-21R-PE (Clone: eBio4A9) mAbs for IL-21R expression on memory B-cell analysis.

Total splenocytes were prepared and performed for intracellularly staining as described previously [19, 20] and analyzed by flow cytometry. The splenocytes were blocked with anti-CD16/32 mAb, immunostained for surface molecules CD4/CXCR5, fixed (1% paraformaldehyde) and permeabilized (0.5% Tween 20), and intracellularly immunostained for Bcl-6, Bcl-6/IL-21, IL-4, or IFN-*γ*. For Tfh cell analysis, the samples were immunostained with anti-mouse CD4-PECy5 (Clone: GK1.5), CXCR5-FITC (Clone: 2G8), and Bcl-6-PE (Clone: G1191E) mAbs. For cytokines expression in Tfh cells, the samples were immunostained with anti-mouse CD4-PECy5 (Clone: GK1.5), CXCR5-FITC (Clone: 2G8) and Bcl-6-PE (Clone: G1191E), IL-21-APC (Clone: eBio4A9), IL-4-PE (Clone: 11B11), or IFN-*γ*-PE (Clone: XMG1.2) mAbs.

### 2.7. Statistic Analysis

Results are depicted as mean ± standard deviation (SD). Pairwise differences were analyzed by the two-sided Student's *t*-test. For multigroup analysis, ANOVA and the Bonferroni tests were used. Differences are considered significant if *P* < 0.05 and very significant and considered very significant if *P* < 0.01.

## 3. Results

### 3.1. Humoral Immune Responses after Vaccination

**Figure 1:**
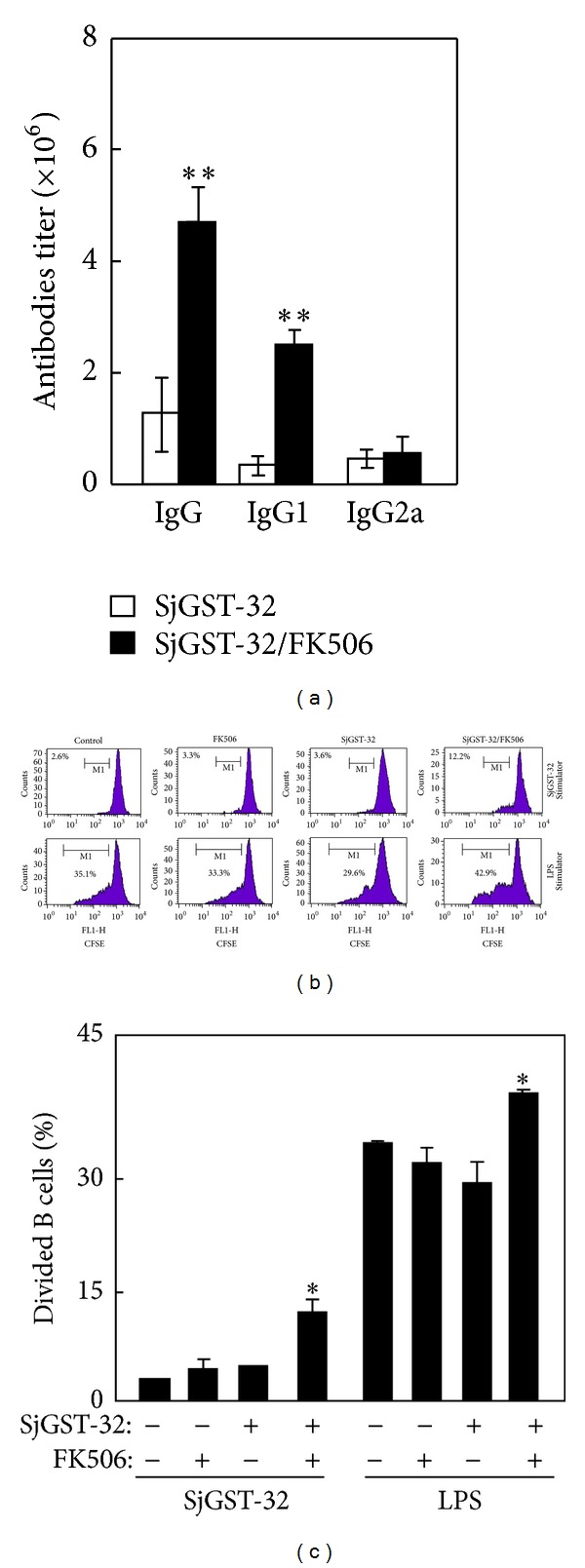
The enhancement of humoral immune response in mice immunized with SjGST-32/FK506. The BALB/c mice were immunized twice with SjGST-32/FK506 in a 2-week interval. (a) Seven days after the second injection, the serum was collected for IgG and IgG1 tests by ELISA. Bar, mean, and SD from 3 independent experiments each using three mice per group. **P < 0.01 indicates statistically very significant between the pairs. (b) Seven days after the second injection, B cells were isolated from splenocytes and the purity of B cells was above 93%. B cells were labeled with CFSE and performed for proliferation assay. The isolated B cells were cultured with SjGST-32 or LPS as stimulator for 72 hours and were analyzed by flow cytometry. Shown in each panel is 1 of at least 3 experiments with similar results. (c) The summary of B-cell proliferation; bar, mean, and SD from 3 independent experiments each using at least three mice per group (*n* = 3). For statistical analysis, mice immunized with SjGST-32/FK506 were compared with those in other groups and ANOVA was used; *P < 0.05. **Indicates that the difference is very significant at the 0.01 level

We have previously shown in mice that FK506 as an adjuvant promotes humoral immune responses to immunogens. Here, to evaluate the humoral immunity induced by the recombinant protein SjGST-32 combined with FK506 as the adjuvant (SjGST-32/FK506), mice were immunized with the combination and serum was later collected for ELISA tests. The antibody titers of total IgG, IgG1 were defined as the highest dilution that gave an above 2 : 1 ratio between testing serum and the naive negative control. The IgG titer of the mice immunized with SjGST-32/FK506 was higher than that of the mice immunized with SjGST-32 alone (Figure 1(a)), which was consist with the known adjuvant effect of FK506 for OVA [21]. Similar difference was seen in the IgG1 titer as well, but not in IgG2a (Figure 1(a)), which demonstrated that FK506 enhanced SjGST-32-specific humoral immunity. 

Since B cells were activated and proliferated after antigen stimulation, to assess the proliferative ability of the B cells in immunized mice, splenocytes were prepared and B cells were purified via positive selection by magnetic cell sorting. The purified B cells were labeled with CFSE and added into 96-well plates for a B cell proliferation assay with SjGST-32 protein or LPS as stimulators. The samples were cultured for 3 days at 37°C and analyzed by flow cytometry. The proliferative activity to SjGST-32 of B cells in mice immunized with SjGST-32/FK506 significantly increased compared with other groups. Furthermore, LPS-stimulated B cells from mice immunized with SjGST-32/FK506 expanded significantly more than other groups (Figures 1(b) and 1(c)).

### 3.2. The Induction of Tfh Cells after Vaccination

**Figure 2:**
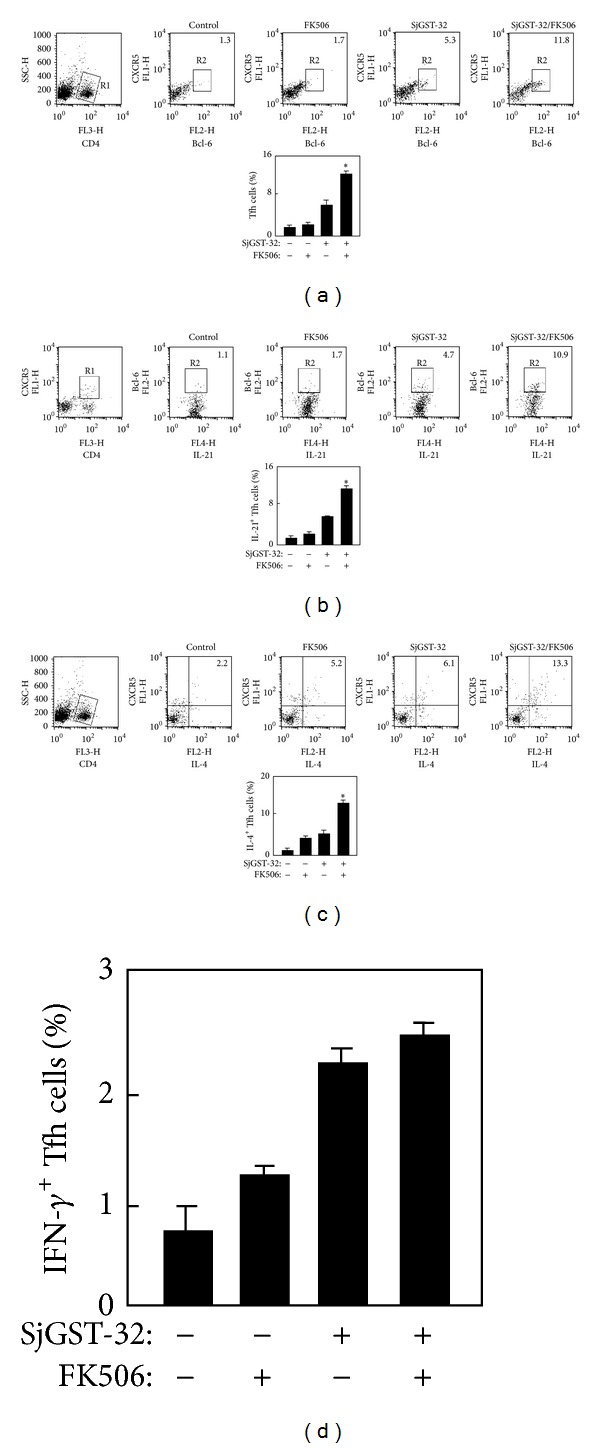
The induction of Tfh cells in mice immunized with SjGST-32/FK506. BALB/c mice were immunized twice with SjGST-32/FK506 in a 2-week interval. Three days after the second injection, the splenocytes were prepared as described in Section 2. (a) The CD4^+^ T cells were gated for analysis of Tfh cells and the data were shown as the percentage of Tfh cells (CD4^+^CXCR5^+^Bcl-6^+^) in total CD4^+^ T cells. The graphic result of the percentage of Tfh cells was shown. (b) The CD4^+^CXCR5^+^ T cells were gated for analysis of IL-21 expression in Tfh cells and the data are shown as the percentage of IL-21^+^ (CD4^+^ CXCR5^+^Bcl-6^+^IL-21^+^) cells in total Tfh cells (CD4^+^CXCR5^+^Bcl-6^+^) and the expression of IL-21 from Tfh cells. The samples were analyzed by flow cytometry. Shown in each panel is 1 of at least 3 experiments with similar results. The graphic result of the percentage of IL-21^+^Tfh cells was shown. (c) The CD4^+^ T cells were gated for analysis of IL-4 level from Tfh cells (CD4^+^CXCR5^+^IL-4^+^). The graphic result of the percentage of Tfh cells was shown. Shown in each panel is 1 of at least 3 experiments with similar results. The graphic result of the percentage of IL-4^+^Tfh cells was shown. Bar, mean, and SD from 3 independent experiments each using three mice per group. For statistical analysis, mice immunized with SjGST-32/FK506 were compared with those in other groups and ANOVA was used; *P < 0.05. (d) The graphic result of the percentage of IFN-*γ* ^+^Tfh cells was shown; bar, mean, and SD from 3 independent experiments each using at least three mice per group (*n* = 3). For statistical analysis, mice immunized with SjGST-32/FK506 were compared with those in other groups and ANOVA was used; *P < 0.05.

Since Tfh cells are required for B-cell activation and proliferation, the induction of Tfh cells was checked in immunized mice. The splenocytes of mice immunized with SjGST-32/FK506 were prepared and immunostained with anti-CD4, anti-CXCR5, anti-Bcl-6, or anti-IL-21 antibodies for flow cytometry analysis. The percentage of CD4^+^CXCR5^+^Bcl-6^+^ Tfh cells (Figure 2(a)) and also the IL-21 producing (CD4^+^Bcl-6^+^IL-21^+^) Tfh cells (Figure 2(b)) of the mice immunized with SjGST-32/FK506 were doubly higher than that of mice immunized with SjGST-32 alone. There were no differences in Th1 (CD4^+^IFN-*γ* ^+^), Th2 (CD4^+^IL-4^+^), Treg (CD4^+^Foxp3^+^), or Th17 (CD4^+^IL-17^+^) between the two groups (data not shown). For immunization with helminth antigens, Tfh cell differentiation was stimulated from Th2 cells. Cytokines such as IL-4 were used as a robust marker of Tfh cells responding to the helminth antigen [2]. To check the expression of cytokines in Tfh cells, splenocytes were intracellularly immunostained with anti-CD4, anti-Bcl-6, IL-4, or IFN-*γ* antibodies for flow cytometry analysis. The number of IL-4 producing (CD4^+^Bcl-6^+^IL-4^+^) Tfh cells of mice immunized with SjGST-32/FK506 was significantly higher than that of control groups (Figure 2(c)) while there was no difference in the number of IFN-*γ* producing (CD4^+^Bcl-6^+^ IFN-*γ* ^+^) Tfh cells among the groups (Figure 2(d)).

### 3.3. Activation of GC B Cells by IL-21 after Vaccination

**Figure 3:**
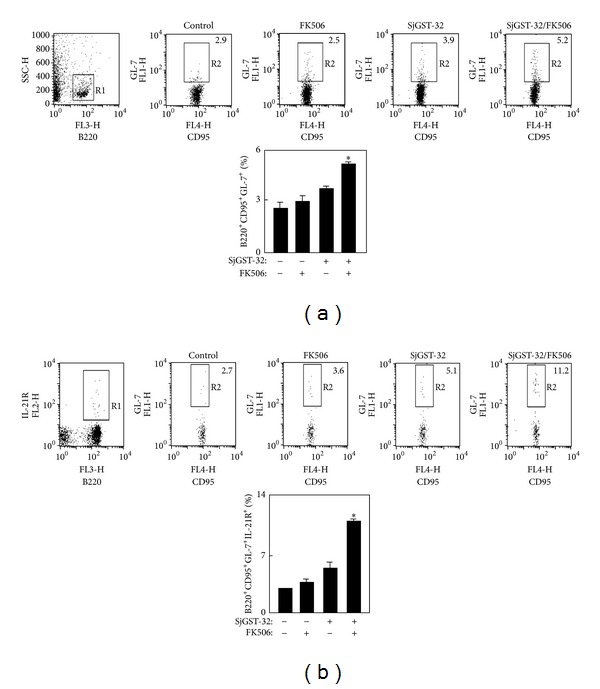
The IL-21R expression of GC B cells in mice immunized with SjGST-32/FK506. The BALB/c mice were immunized twice with SjGST-32/FK506 in a 2-week interval. (a) Seven days after the second injection, the splenocytes were prepared and stained with anti-mouse B220, GL-7, and CD95 mAbs. The samples were analyzed by flow cytometry. The B220^+^ cells were gated for analysis of GCs B cells and the data are shown as the percentage of GC B cells (B220^+^CD95^+^GL-7^+^) in total B cells (B220^+^). Shown in each panel is 1 of at least 3 experiments with similar results. The graphic result of the percentage of GC B cells was shown. (b) Seven days after the second injection, the splenocytes were prepared and stained with anti-mouse B220, IL-21R Abs, GL-7C, and CD95 mAbs. The B220^+^IL-21R^+^ cells were gated and the data are shown as the percentage of IL-21R expression (B220^+^IL-21R^+^CD95^+^GL-7^+^) in total GC B cells (B220^+^CD95^+^GL-7^+^). Shown in each panel is 1 of 3 experiments with similar results. The graphic result of the percentage of IL-21R^+^GC B cells was shown; bar, mean, and SD from 3 independent experiments each using three mice per group. For statistical analysis, mice immunized with SjGST-32/FK506 were compared with those in other groups and ANOVA was used; *P < 0.05.

IL-21, which is expressed strongly by Tfh cells, drives the growth and differentiation of B cells and is essential for GC B cell survival and proliferation by direct action on IL-21 receptor (IL-21R)-expressing B cells [4, 10]. As GC B cells markedly express GL-7 and CD95 [23], the splenocytes were prepared and immunostained with anti-B220/anti-GL-7/anti-CD95 antibodies for GC B cells analysis, or with anti-B220/anti-GL-7/anti-CD95/anti-IL-21R antibodies for IL-21R expression on the GC B cells. The percentage of GC B cells of mice immunized with SjGST-32/FK506 was significantly higher than that of control groups (Figure 3(a)). The IL-21R expression on the GC B cells of mice immunized with SjGST-32/FK506 was also significantly higher than those of control groups (Figure 3(b)). IL-21R expression on Tfh cells was also tested, but there was no difference among the groups (data not shown).

### 3.4. Tfh Cells Stimulated the Formation of Memory B Cells after Vaccination

**Figure 4:**
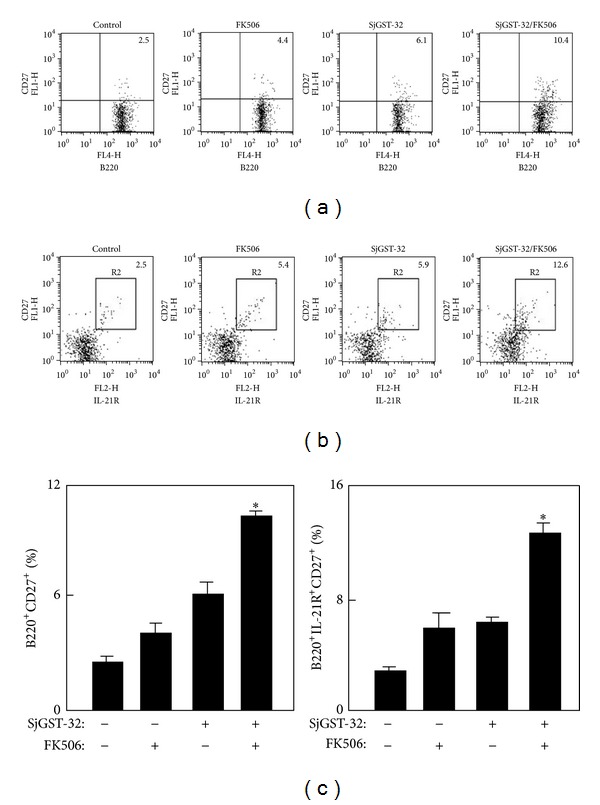
The expression of IL-21R on memory B cells was increased in mice immunized with SjGST-32/FK506. The BALB/c mice were immunized twice with SjGST-32/FK506 in a 2-week interval. Forty-nine days after the second injection, the splenocytes were prepared. (a) The samples were stained with anti-mouse B220-APC and CD27-PE Abs for memory B cells. The B220^+^ cells were gated and the data are shown as the percentage of memory B cells (B220^+^CD27^+^) in total B cells (B220^+^). Shown in each panel is 1 of at least 3 experiments with similar results. (b) The splenocytes were stained with anti-mouse B220, IL-21R, and CD27 Abs for IL-21R expression on memory B cells. The samples were analyzed by flow cytometry. The B220^+^ cells were gated and the data are shown as the IL-21R expression (B220^+^CD27^+^IL-21R^+^) on memory B cells (B220^+^CD27^+^). Shown in each panel is 1 of 3 experiments with similar results; bar, mean, and SD from 3 independent experiments each using at least three mice per group (*n* = 3). For statistical analysis, mice immunized with SjGST-32/FK506 were compared with those in other groups and ANOVA was used; *P < 0.05.

Tfh cells are required for the formation and maintenance of GCs and also for the generation of memory B cells and plasma cells. The memory B cells are essential for vaccines to generate effective antibody responses when encountering the same antigen. To test memory B cells of mice immunized with SjGST-32/FK506, the splenocytes from immunized mice were immunostained with anti-B220, anti-CD27, and IL-21R antibodies for flow cytometry analysis on day 45 after the last vaccination. The number of memory B cells (B220^+^CD27^+^) (Figure 4(a)), and also the IL-21R expression on the memory B cells (B220^+^CD27^+^IL-21R^+^) (Figure 4(b)), of mice immunized with SjGST-32/FK506 was significantly higher than that of control groups, which indicated that adjuvant-induced Tfh cells promoted the generation of memory B cells by the IL-21 pathway.

## 4. Discussion

In this study, we investigated the induction of Tfh cells in mice immunized with a Schistosoma antigen and FK506. We showed that FK506 induced Tfh cells and enhanced B cells function and subsequent IgG responses, especially the IgG1 level. We found that IL-21 was highly expressed on Tfh cells, whereas IL-21R was highly expressed on GC B cells and also memory B cells. Moreover, we also found that IL-4–producing Tfh cells were significantly increased in mice immunized with SjGST-32/FK506, which was consistent with a crucial role for GCs in Tfh cell development in the context of a Th2 response [2]. These results suggest that FK506 as an adjuvant can induce Schistosoma antigen-reactive Tfh cells and IL-21 expression from the adjuvant-induced Tfh, which in turn stimulate B cells function and humoral immune responses. 

Antibody production is critical for pathogen clearance. To most proteins, this type of humoral responses is strictly TD [1]. Vaccination is arguably the best solution to eradicate helminthiases including Schistosoma. Various approaches are in progress recently in order to improve vaccination against Schistosoma, particularly with respect to the choice of a better adjuvant. LMS as adjuvant of DNA vaccine encoding the SjGST-32 gene enhanced both worm killing and disease prevention by the induction of strong Th1 immune responses [16]. 

FK506, known as an immunosuppressant, suppresses the activation of Th1 cells, which produce IL-2 [17]. Recently, evidence has accumulated indicating that FK506 can affect DCs and also Treg cells [18–20]. FK506 combined with experimental autoimmune encephalomyelitis (EAE) DNA vaccine could induce Treg cells [19]. Furthermore, FK506 as an adjuvant for OVA induced Tfh cells has been shown to enhance humoral immune responses [21]. Here, we demonstrated that FK506 as an adjuvant for the Schistosoma recombinant protein induced Tfh cells, a subset of effector T cells that helps the development of Ag-specific B cell responses in GCs. It is clear that Tfh cells depend on expression of CXCR5, Bcl6, and IL-21, which allow their localization in the follicular regions of lymphoid organs and their stable contacts with Ag-primed B cells [2–4]. IL-21 production by Tfh cells is essential for GC B-cell survival and proliferation by direct action on IL-21R expressing B cells. Here, we found that IL-21R expressed on GC B cells in mice immunized with FK506 as adjuvant. Different kinds of antigens can induce variant immune response, and also for different types of Th cells. SjGST-32 as an antigen could stimulate immune response to Schistosoma. When mice were coimmunized with SjGST-32 and FK506, Tfh cells were induced, but not Treg cells.

IL-4, as a cytokine produced by Th2 cells, plays a crucial role in promoting host survival during infection with parasitic helminthes. Importantly, Th2 responses are associated with the development of strong antibody responses, particularly IgG1, which in certain helminth infections are implicated in resistance to reinfection. King and Mohrs reported that. During helminth infection, Tfh cells in reactive lymph nodes produced IL-4 while its deletion resulted in defective B-cell expansion and maturation [2]. Zaretsky et al. reported that after helminth infection, or immunization with helminth antigens, IL-4 expressing Th cells displayed the Tfh markers CXCR5, Bcl-6, and IL-21 [24]. Tfh cells have been shown to make the Th2 signature cytokine IL-4 [6]. It is thus possible that a relationship exists between Th2 and Tfh cells.

Here, we also found that mice immunized with SjGST-32/FK506 induced IL-4 producing Tfh cells.

Tfh cells are required for the formation and maintenance of GCs and generation of most memory B cells and plasma cells. The products of the GC reaction are memory B cells and high-affinity plasma cells. There are no known signals that directly drive GC B cell to memory B-cell differentiation [25]. Memory B-cell differentiation is much less clear. Here, we found that IL-21R was highly expressed on memory B cells, which indicated that IL-21 produced by adjuvant-induced Tfh cells might stimulate the formation of memory B cells. 

In summary, our results demonstrate, for the first time, that FK506 as an adjuvant for *Schistosoma japonicum* recombinant protein augmented the induction of Tfh cells that expressed IL-21 and IL-4 and produced memory B cells. Furthermore, the protective immune response of SjGST-32/FK506 will be studied in the future. Since FK506 is already an approved clinic drug, its use as adjuvant at low dosage for *Schistosoma japonicum *recombinant protein vaccination should be safe enough to advance to clinical trials to further examine its potential as a human adjuvant. A more general application of FK506 as adjuvant for induction of Tfh cells and memory B cells may also be explored for other protein vaccines to control parasitic helminth. The effect and mechanism of FK506 as adjuvant of helminth antigen need further exploring.
